# Dual localization of wild-type myocilin in the endoplasmic reticulum and extracellular compartment likely occurs due to its incomplete secretion

**Published:** 2009-03-13

**Authors:** Seongsoo Sohn, Myung Kuk Joe, Tae Eun Kim, Ji-eun Im, Young Ran Choi, Hwayong Park, Changwon Kee

**Affiliations:** 1 Department of Ophthalmology, Samsung Medical Center, Sungkyunkwan University School of Medicine, Seoul, South Korea; 2 Center for Clinical Research, Samsung Biomedical Research Institute, Sungkyunkwan University School of Medicine, Seoul, South Korea; 3 Department of Medical Research, Korea Institute of Oriental Medicine, Daejeon, South Korea

## Abstract

**Purpose:**

Wild-type myocilin is known to be secreted extracellularly, but a significant amount of the protein is also present in the endoplasmic reticulum (ER). The present study was undertaken to address whether intracellular myocilin is a true ER resident protein.

**Methods:**

Human wild-type myocilin was adenovirally expressed in human trabecular meshwork cells, and general characteristics of both intracellular and extracellular myocilins including molecular weight, pI, glycosylation state, and cleavage site of the signal peptide were examined by biochemical analyses. Topology and decay kinetics of myocilin were also examined by protease protection assay and pulse chase analysis, respectively. The expression pattern and cytopathic effect of myocilin were analyzed in individual cells by immunocytochemistry.

**Results:**

Intracellular myocilin were very similar to secreted myocilin in characteristics such as molecular weight, pI, glycosylation state, and cleavage site of the signal peptide. The intracellular protein was found to be present in the lumen of the ER where it appeared to be retained without further export to the Golgi apparatus. The kinetics of myocilin turnover clearly showed that it was intrinsically a very stable but incompletely secreted protein. The expression of myocilin was confined to a subset of cells and accompanied by the upregulation of a 78 kDa glucose-regulated protein, suggesting that it was not properly folded or processed in the ER.

**Conclusions:**

Based on these findings and the fact that myocilin has no known ER retention signals, the ER localization of wild-type myocilin is likely a consequence of its incomplete secretion due to its misfolding.

## Introduction

Primary open-angle glaucoma (POAG) characterized by the gradual death of retinal ganglion cells is the most common form of glaucoma that develops in up to 2% of people older than 40 years of age [1-3]. The major risk factor for POAG is an elevated intraocular pressure (IOP), which is consequent to the decreased aqueous outflow through the trabecular meshwork (TM), a reticulated tissue at the junction of the cornea and iris through which approximately 90% of aqueous humor (AH) exits [1,2].

*MYOC*, encoding a glycoprotein termed myocilin, was the first gene linked to POAG [4]. Myocilin is an acidic protein with a molecular weight of 55 kDa and comprises two major domains, an NH_2_-terminal coiled coil domain with a leucine zipper-like motif and a COOH-terminal olfactomedin-like domain. The NH_2_-terminal domain has 77.6% homology with the myosin heavy chain of *Dictyostelium discoideum* and shares 25% identity with the cardiac beta myosin heavy chain [5,6]. These analogies have led to the protein name myocilin [6].

Currently, up to 70 different disease-causing *MYOC* mutations have been described in patients with autosomal dominant juvenile open-angle glaucoma and some forms of adult-onset POAG [7-9]. Fingert et al. [10] termed POAG caused by *MYOC* mutations “myocilin glaucoma”. It has been estimated that myocilin glaucoma accounts for about 3%−4% of all cases of POAG [7-9].

Although little is known about the functions of myocilin, it has been suggested that myocilin mutations in humans likely act through a gain of function [11-13]. Biochemical and cell biological studies gave supportive results that myocilin mutants are not secreted but retained in the endoplasmic reticulum (ER) [14-19]. It has been reported that retention of myocilin mutants in the ER initially leads to ER stress and eventually to the death of human trabecular meshwork (HTM) cells [16-18]. Pathologically, cell death is a critical component of gain of function because it inevitably leads to diminution in the cell number of the TM, a hallmark of POAG [20,21]. However, it is uncertain whether such a scenario also occurs in wild-type myocilin.

There is no doubt that wild-type myocilin is normally secreted because numerous studies have reported its detection in culture media of various cell lines in vitro [14-19,22-27] and in the AH of several species including humans in vivo [15,26,28,29]. It should be noted, however, that a significant amount of wild-type myocilin is also present intracellularly [14,15,19,22-27], especially in the ER [16-18]. Although examples are being found of proteins that are resident and function in more than one cellular compartment, an ER location is unusual for secretory proteins. Generally, proteins retained in the ER are categorized into two types, the misfolded or incompletely assembled proteins and the ER resident proteins. Presently, there is no evidence to suggest that myocilin is an actual ER resident protein.

In this study, we characterized both the intracellular and extracellular myocilins in an effort to better understand the characteristics of myocilin in the ER. Herein, we present evidence that does not support the hypothesis that intracellular myocilin is a true ER resident protein.

## Methods

### Cell culture

HTM cells derived from the normal eyes of a 27-year-old female donor were a generous gift from Paul L. Kaufman (University of Wisconsin, Madison, WI). Human embryonic kidney 293A cells were purchased from Qbiogene (Carlsbad, CA). These cells were cultured in Dulbecco’s modified Eagle’s medium (DMEM; Life Technologies, Rockville, MD) supplemented with 10% fetal bovine serum (FBS; Life Technologies) and antibiotics (100 units/ml penicillin, 100 μg/ml streptomycin, and 20 μg/ml gentamicin) at 37 °C in a humidified chamber with 5% CO_2_.

### Viral constructs

Replication-deficient recombinant adenoviruses expressing human wild-type myocilin tagged with the FLAG epitope (Asp-Tyr-Lys-Asp-Asp-Asp-Asp-Lys) at its COOH-terminus (Ad-myocilin-FLAG) or at its NH_2_-terminus (Ad-FLAG-myocilin) were generated using the AdEasy^TM^ system (Qbiogene) according to the manufacturer’s protocol. To construct a vector encoding myocilin with a FLAG tag at its COOH-terminus, the open reading frame of myocilin without the stop codon was excised from the pΔACMV-wild-type myocilin-GFP vector that encodes a fusion protein joining human myocilin to the NH_2_-terminus of the green fluorescence protein (GFP) [16]. The excised myocilin cDNA was then inserted into the BamH 1 and Age 1 sites of the pShuttle-CMV vector (Qbiogene) that was previously modified by inserting an adaptor containing an Age 1 site and the sequences for the FLAG epitope. The adaptor was generated by annealing primers 5′-TCG AGA CCG GTC GAT TAC AAG GAT GAC GAC GAT AAG TAG-3′ and 5′-CTA GCT ACT TAT CGT CGT CAT CCT TGT AAT CGA CCG GTC-3′. To construct a vector encoding myocilin with a FLAG tag at its NH_2_-terminus, cDNA for myocilin was amplified by polymerase chain reaction (PCR) using the pΔACMV-wild-type myocilin-GFP vector as a template with primers 5′-NNN GGA TCC AGG AAG CCT CAC CAA GCC-3′ and 5′-NNN AAG CTT CAC ATC TTG GAG AGC TT-3′ and then cloned into the BamH 1 and Hind III sites of the pShuttle-CMV vector that was previously adapted by inserting the sequences for the FLAG epitope. The region encoding the FLAG epitope was amplified by PCR from the pCMV-Tag 2B vector (Stratagene, La Jolla, CA) using primers 5′-NNN AGA TCT GCC ACC ATG GAT TAC AAG-3′ and 5′-NNN AAG CTT GAT ATC GAA TTC CTG CAG-3′. The resulting shuttle vectors encoding the FLAG-tagged myocilins were linearized with Pme 1 and co-transformed into BJ5183 along with pAdEasy-1, a plasmid containing the adenovirus serotype 5 genome with deletions in the E1 and E3 regions. The recombinant adenoviral constructs were selected with kanamycin, cleaved with Pac 1, and then transfected into 293A cells to produce viral particles. Viral titers were determined by limiting dilution on 293A cells, and the absence of *E1a* in the viral constructs was confirmed by PCR. Other adenoviruses expressing GFP alone (Ad-GFP), myocilin with its COOH-terminus tagged with GFP (Ad-myocilin-GFP), a myocilin mutant with an Y437H mutation (Ad-Y437H myocilin-GFP), and stromelysin (Ad-stromelysin-GFP) have been previously described [16,17]. Unless specified otherwise, viral transduction was performed for 2 h at a multiplicity of infection (MOI) of 10 plaque forming units (pfu) per cell, and the transduced cells were further cultured for 48 h.

### Western blot analysis

Protein samples were mixed with Laemmli sample buffer, boiled for 5 min, and subjected to sodium dodecyl sulfate polyacrylamide gel electrophoresis (SDS–PAGE). The resolved proteins were transferred to a nitrocellulose membrane and blocked with 5% skim milk in Tris buffered saline (TBS) containing 0.05% Tween-20 (TTBS) overnight. The membrane was incubated for 2 h with anti-myocilin antibody diluted 1,000 fold, monoclonal anti-FLAG antibody (Sigma, St Louis, MO) diluted 2,000 fold, or monoclonal anti-calnexin antibody (BD Transduction Laboratories, Lexington, KY) diluted 500 fold in TTBS. The anti-myocilin antibody was raised in rabbits against the peptide sequence Arg-Leu-Arg-Gln-Glu-Asn-Glu-Asn-Leu-Ala-Arg-Arg that corresponds to positions 158–169 of human myocilin. After washing three times with TTBS, the membrane was reacted for 2 h with peroxidase-conjugated anti-mouse or anti-rabbit antibodies (Amersham, Buckinghamshire, UK) diluted 2,000 fold in TTBS. The membrane was washed three times and then probed with the chemiluminescence kit (Amersham) using the manufacturer’s protocol.

### Enzymatic deglycosylation

For endoglycosidase H (Endo H) and N-glycosidase F (PNGase F; New England Biolabs, Ipswich, MA) digestions, samples were denatured with 0.5% SDS and 1% β-mercaptoethanol at 100 °C for 10 min. For the Endo H digestion, the denatured samples were mixed with 50 mM sodium citrate (pH 5.5) plus 500 units of the enzyme. For the PNGase F treatment, the samples were suspended in 50 mM sodium phosphate buffer (pH 7.5) containing 1% NP-40 as well as 500 units of the enzyme. For endoglycosidase D (Endo D; Seikagaku, Tokyo, Japan) digestion, cell lysates prepared in lysis buffer composed of 50 mM Tris (pH 7.5), 150 mM NaCl, 1 mM EDTA, 1% Triton X-100, and 1X protease inhibitor (Roche, Indianapolis, IN) were mixed with citrate-phosphate buffer (pH 6.5) to a final concentration of 150 mM and 10 milliunits of the enzyme was added. All deglycosylation reactions were done at 37 °C for 1 h.

### Amino acid sequencing

Confluent HTM cells in a 100 mm culture dish were transduced with Ad-myocilin-FLAG, washed thoroughly with serum-free DMEM, and then further cultured with the serum-free media. Forty-eight hours after transduction, the media (5 ml) was collected, spun at 14,000x g for 20 min to eliminate insoluble materials, and then concentrated 25 fold (final volume of 200 μl) using a centrifugal filter device (Centricon YM-30; Millipore, Corporation, Bedford, MA). The cells were lysed with 1 ml lysis buffer, spun briefly, and then 200 μl of 50% anti-FLAG M2 affinity gel slurry (Sigma) was added. The mixtures were spun again, and the pellet was washed twice with TBS. Protein elution was performed by a competition with 200 μl of 450 ng/μl 3X FLAG (Sigma). The purified proteins and concentrated media were subjected to SDS–PAGE, and the resolved proteins were transferred to a polyvinylidene fluoride membrane and stained with a Coomassie brilliant blue R250. For NH_2_-terminal amino acid sequencing via the Edman degradation method, the Coomassie stained proteins were excised from the membrane and installed into the blot cartridge of a Perkin-Elmer protein sequencer (Molde 491A; Perkin-Elmer, Foster City, CA).

### Two-dimensional gel electrophoresis

HTM cells transduced with Ad-myocilin-FLAG in a 100 mm dish were cultured with serum-free media. After 48 h, the media (5 ml) was collected and added with 50 μl of Triton X-100 and 100 μl of 50X protease inhibitor while cells were lysed with 5 ml of the medium containing 1% Triton X-100 plus 1X protease inhibitor. Both cell lysates and media were then precipitated with the 2D Clean-Up Kit (Amersham) and solubilized in 5% IPG (immobilized pH gradient) buffer containing 8 M urea, 2% 3-[(3-cholamidopropyl)dimethylammonio]-1-propanesulfonate (CHAPS), and 60 mM DTT. The dissolved proteins were applied on 17 cm IPG (pH 4–7) linear strips (Amersham) and allowed to be rehydrated at ambient temperature overnight. Isoelectric focusing started at 300 V for 1 hour, continued with a gradient at 1,000 V for 1 h and 8,000 V for 6 h, and finished at a total of 49,300 Vh. The focused strips were immersed in succession with the equilibration buffer (50 mM Tris, pH 8.8; 6 M urea; 2% SDS; 130 mM DTT; 20% glycerol) and the same buffer supplemented with 135 mM iodoacetamide instead of DTT for 15 min each. The strips were washed with running buffer (25 mM Tris, 192 mM glycine, 0.1% SDS, pH 8.3), secured on top of a 13% acrylamide gel, and then electrophoresed at 100 V until the bromophenol blue reached the bottom of the gel. The resolved gels were then processed for western blot analysis, and the proteins were probed with monoclonal anti-FLAG antibody.

### Metabolic labeling and immunoprecipitation

HTM cells in a 24 well culture plate were transduced with Ad-myocilin-GFP or Ad-stromelysin-GFP. Twenty-four hours post-transduction, the cells were starved for 90 min in methionine-free DMEM, pulse-labeled for 30 min with 50 μCi [^35^S]methionine (Amersham), and chased with DMEM containing 10 mM methionine in the presence or absence of acetyl-leucyl-leucyl-norleucinal (ALLN, 10 μg/ml; Sigma). After the appropriate duration of chase, the media was harvested, and the cells were lysed with 0.3 ml of immunoprecipitation buffer (50 mM Tris, pH 7.5; 150 mM NaCl; 1% NP-40; 10% glycerol; 1X protease inhibitor mixture). The media and cell lysates were spun at 14,000x g for 20 min, and 100 μl of the supernatants were mixed with 2 μg of monoclonal anti-GFP antibody (Sigma) and 20 μl of 50% protein A-agarose slurry (Santa Cruz Biotechnologies, Santa Cruz, CA). The mixtures were briefly centrifuged, and the pellet was washed twice with immunoprecipitation buffer followed by washing once with phosphate buffered saline (PBS). The pellets were suspended with 20 μl of 1X Laemmli sample buffer, boiled, and resolved on a 10% polyacrylamide gel. The gel was dried, and autoradiography was performed.

### Protease protection assay

Sealed microsomal membrane vesicles were prepared from ~10^7^ transduced HTM cells using an Endoplasmic Reticulum Isolation Kit (Sigma). The crude microsomal fraction was pelleted by centrifugation at 100,000x g for 60 min and suspended in 0.2 ml of 10 mM 4-(2-hydroxyethyl)piperazine-1-ethanesulfonic acid (HEPES; pH 7.8) containing 250 mM sucrose, 25 mM KCl, 1 mM ethylene glycol-bis(2-aminoethylether)-N,N,N',N'-tetraacetic acid (EGTA), and 1X protease inhibitor. Aliquots of the membrane suspension (15 μl) equivalent to ~20 μg of protein were incubated in a total volume of 18 μl with varying amounts of freshly prepared proteinase K (final concentration ~0.0-0.1 mg/ml) in the presence and absence of 1% (v/v) Triton X-100 for 30 min on ice. The reactions were stopped by the addition of 2 μl of 200 mM phenylmethylsulfonyl fluoride and 7 μl of 4X Laemmli sample buffer after which the samples were boiled and immediately subjected to western blot analysis.

### Immunocytochemistry

Transduced HTM cells in 4 well chamber slides were washed once with PBS, fixed for 20 min with 4% paraformaldehyde, and permeabilized for 5 min with 0.5% Triton X-100. The cells were blocked with 2% bovine serum albumin (BSA) for 1 h and incubated for 2 h with fluorescein isothiocyanate (FITC)-conjugated or non-conjugated monoclonal anti-FLAG antibody (Sigma) diluted 200 fold or monoclonal anti-78 kDa glucose-regulated protein (GRP78) antibody (BD Transduction Laboratories) diluted 100 fold. The cells were washed, reacted for 2 h with tetramethylrhodamine isothiocyanate (TRITC)-conjugated anti-mouse immunoglobulin antibody (Zymed, South San Francisco, CA) diluted 50 fold. After the nuclei were counterstained for 20 min with 4’, 6’-diamidino-2-phenylindole (DAPI), the cells were viewed under a fluorescence microscope with the appropriate filter sets and a 400X objective.

### Labeling of adenovirus

Prior to fluorescence labeling, recombinant adenovirus was purified by double cesium chloride gradient centrifugation according to the recommendations of the AdEasy^TM^ system. The purified virus was then labeled with a chloromethylbenzamido derivative of Dil (CM-Dil; Molecular Probes, Eugene, OR) following the manufacturer’s guidelines with slight modifications. Briefly, 20 μl of the viral stock (~10^11^ pfu/ml) was diluted with an equal volume of Hank’s balanced salt solution (HBSS) and mixed with 40 μl of 100 mM CM-Dil prepared in HBSS. The viral mixture was incubated for 5 min at 37 °C and for an additional 15 min at 4 °C. Gel filtration chromatography with a G-50 Sephadex column (Roche) was used to isolate the fluorescently labeled viral particles.

## Results

### Intracellular myocilin is identical with its secreted form

As a first step toward elucidating the mechanism by which myocilin is localized to the ER, we attempted to find evidence for the presence of myocilin isoforms because multi-compartmentalized proteins are generally present as isoforms with different topogenic sequences that are specific for each compartment [30]. We constructed a human wild-type myocilin carrying the FLAG epitope at its COOH-terminus (myocilin-FLAG), expressed the construct in the HTM cells by using adenoviral vector, and then examined the previously characterized myocilin expression in the ocular cells that are relevant to glaucoma research.

**Figure 1:**
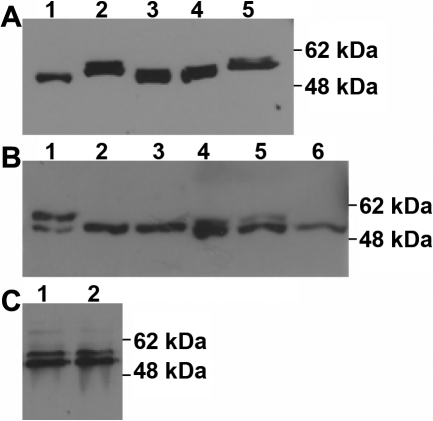
Electrophoretic mobility and glycosylated status of intracellular and extracellular wild-type myocilins. HTM cells were plated into a 24 well plate, grown to 70% confluence, and transduced for 2 h with Ad-myocilin-FLAG at an MOI of 10 pfu. After 48 h, the cells and culture media were harvested and the myocilin in the samples were analyzed by western blot using polyclonal anti-myocilin antibody. **A**: Cells and culture media were harvested together (lane 1), or cells and culture media were separately harvested (lane 2 and 3), or cells were replenished after culture with fresh culture media and then cells and culture media were harvested together (lane 4), or cells were cultured with serum-free culture media and then the conditioned media was harvested (lane 5). Cells were harvested with 0.4 ml of 1X Laemmli sample buffer whereas culture media (0.3 ml) was harvested with 0.1 ml of 4X Laemmli sample buffer. **B**: Myocilin in cells (lane 2 and 3) or media (lane 5 and 6) was treated with Endo H (lane 2 and 5) or PNGase F glycosidase (lane 3 and 6) as described in Methods. Undigested myocilins in cells (lane 1) and culture media (lane 4) served as controls. **C**: Myocilin in cells was either treated (lane 1) or not treated (lane 2) with Endo D glycosidase.

It has been reported that in the standard conditions of SDS–PAGE, the mobility of secreted myocilin is slightly increased compared to intracellular myocilin, although both myocilins are resolved as a doublet at a molecular weight of approximately 55−57 kDa (compare lane 2 with lane 3 in Figure 1A) [15,24]. The different mobility of the intracellular myocilin compared to the extracellular myocilin implies different molecular weights, which can be accepted as evidence for the presence of myocilin isoforms. However, the mobility of the intracellular myocilin also increased when cell lysates were added with culture media containing FBS (lane 4 in Figure 1A). By contrast, the mobility of the secreted myocilin decreased when cells were cultured without FBS (lane 5 in Figure 1A) or only increased mobility was observed when the cell lysate and medium were combined (lane 1 in Figure 1A). Therefore, the fast mobility of the secreted myocilin must have been an artifact.

It has also been reported that the upper bands of the myocilin doublet correspond to the glycosylated forms of the lower bands [14,19,22,25], but a recent study showed that myocilin is not glycosylated [27]. To clarify this discrepancy, we reexamined the effect of deglycosylation on the doublet. The treatment of Endo H resulted in a shift of the upper band into the lower band in the intracellular myocilin but not in the extracellular myocilin (lane 2 and 5 in Figure 1B) while the treatment with PNGase F induced a shift in both the intracellular and extracellular myocilins (lane 3 and 6 in Figure 1B). Endo H removes *N*-glycans of a high mannose type whereas PNGase F completely digests all *N*-linked oligosaccharides. Thus, these results support the earlier finding that myocilin can be glycosylated in the ER and then further processed in the Golgi apparatus. We also examined the effect of Endo D on the doublet to determine whether intracellular myocilin is statically retained in the ER without recycling or retrieved from the *cis*-Golgi. Endo D is a specialized carbohydrate cleaving enzyme that distinguishes glycoproteins that are never exported out of the ER from those entering the *cis*-Golgi [31]. There was no shift in the upper band of the intracellular myocilin after treatment with Endo D (Figure 1C), revealing that at least the glycosylated myocilin is localized to the ER by a retention mechanism.

**Figure 2:**
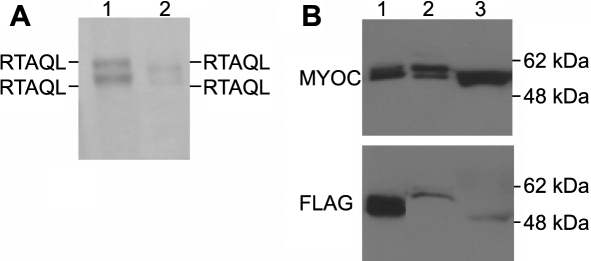
Characterization of the signal sequence cleavage of myocilin. **A**: HTM cells were transduced with Ad-myocilin-FLAG and myocilin in cells (lane 1), and culture media (lane 2) were separated by SDS–PAGE, transferred to the membrane, and loaded into a protein sequencer. All myocilin bands yielded the same results with respect to their NH_2_-terminal amino acid sequences. **B**: HTM cells were transduced with Ad-FLAG-myocilin and myocilin in the cells (lane 2), and culture media (lane 3) was detected by western blot analysis using anti-myocilin antibody (upper panel) or anti-FLAG antibody (lower panel). Myocilin-FLAG (Figure 1A, lane 2) was included as a control (lane 1).

We determined the NH_2_-terminal amino acid sequence of myocilin and obtained the following sequence: Arg-Thr-Ala-Gln-Leu, which matches the residues 33–37 of myocilin where the predicted cleavage site of the ER targeting signal sequence of myocilin starts [32]. We found no differences in the sequences between intracellular and extracellular myocilin and between the upper and low bands of both of the myocilins (Figure 2A), demonstrating that the signal sequence is cleaved off all forms of myocilin. Consistent with these results, myocilin with its NH_2_-terminus tagged with FLAG was detected by anti-myocilin antibody but not by anti-FLAG antibody in both the cell lysate and culture medium. However, myocilin-FLAG was clearly detected by anti-FLAG antibody (Figure 2B).

**Figure 3:**
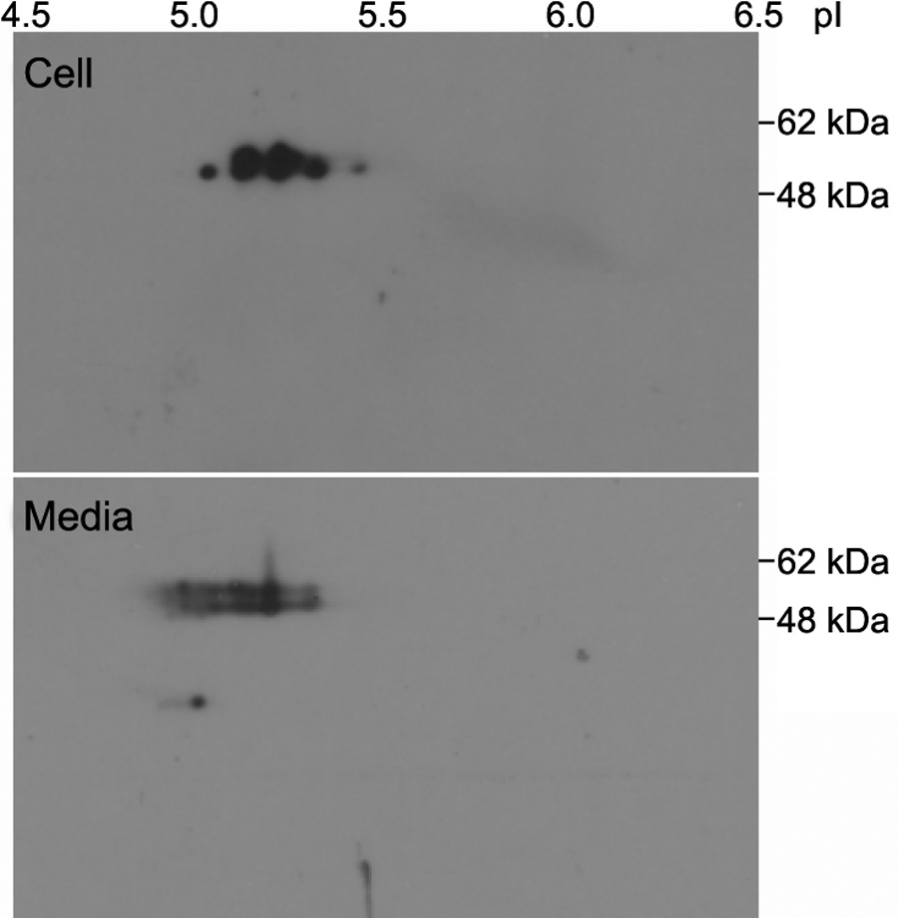
Two dimensional analysis of myocilin expression. Myocilin isoforms in cell lysate (upper panel) and culture medium (lower panel) from HTM cells transduced with Ad-myocilin-FLAG were first separated by their isoelectric points and then resolved according to their molecular weights. Myocilin detection was performed by western blot analysis using anti-FLAG antibody. This experiment was performed two times, and representative gels are shown.

Previously, two dimensional (2D) PAGE analysis of myocilin expression showed that multiple isoforms could be detected in cell lysates of human optic nerve head cells or in the media of human lamina cells as well as in cell lysates and media of dexamethasone-treated HTM cells [22,23,33]. Because 2D-PAGE is the only method used to describe myocilin isoforms in the literature, we also used it to identify myocilin isoforms that are specific to the intracellular or extracellular compartments. To precisely compare the myocilin isoforms in the cell lysate with those in the culture media, we simultaneously performed all experimental procedures including the sample preparations and the electrophoresis. In agreement with previous results, western blot analysis following 2D-PAGE of cell lysates showed five to six different myocilin isoforms ranging in pI from 5.0 to 5.4 and molecular weight from 55 to 57 kDa (Figure 3, upper panel) whereas the myocilin in concentrated media was separated as multiple isoforms with pI ranging from 4.9 to 5.3 and molecular weight from 55 to 57 kDa (Figure 3, lower panel). It is important to point out that a significant amount of the myocilin isoforms with same mass and pI were found in both the cell lysate and medium, although it is evident that about half of the myocilin isoforms in the culture medium were more acidic than those in the cell lysate. In addition to the preceding data, these results consistently showed that there were no myocilin isoforms found only in the ER or extracellular compartment.

### Myocilin is not completely secreted

**Figure 4:**
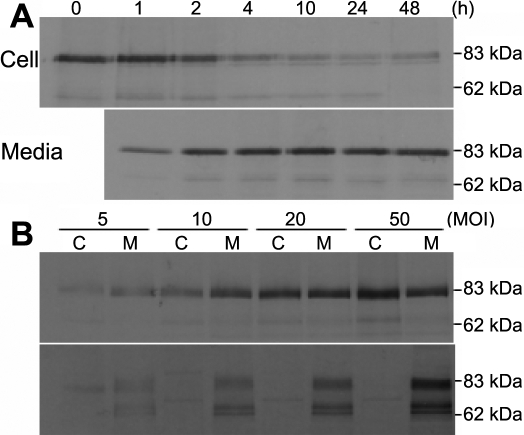
Time-course analysis of the cellular trafficking of myocilin. **A**: HTM cells transduced with Ad-myocilin-GFP at an MOI of 10 pfu were pulse labeled with [^35^S]methionine and chased for the times indicated. After chase, the GFP fusion protein in the equal amount of cell lysates (~5 μg/well) and culture media were immunoprecipitated with anti-GFP antibody, resolved on SDS–PAGE, and then subjected to autoradiography. Each experiment was performed three times with similar results. **B**: HTM cells transduced with Ad-myocilin-GFP (upper panel) or Ad- stromelysin-GFP (lower panel) at the indicated MOI were pulse labeled and chased for 4 h. The cells (C) and culture media (M) were separately harvested and processed the same as in **A**. Each experiment was performed two times with identical results. The bands resolved at approximately 68 kDa might be degraded products of stromelysin-GFP.

The absence of a difference between the intracellular and extracellular myocilins led us to assume that the intracellular myocilin is a protein that is embarking on the secretory pathway. To test this possibility, we examined the trafficking of myocilin with its COOH-terminus tagged with GFP (myocilin-GFP). Although most of the fusion protein was secreted as early as 1 h after chase and completed its secretion approximately 2 h after chase, detectable amounts of the initial pulse remained in the cells even 48 h after chase. In addition, there was no intracellular decay observed throughout the chase period (Figure 4A). Moreover, the treatment with the proteasomal inhibitor, ALLN, had no effect on the decay (data not shown). Taken together, these results suggest that myocilin is intrinsically very stable but an incompletely secreted protein.

To further explore the export of myocilin from HTM cells, we repeated the above experiment but varied the level of myocilin expression. At lower expression levels as in Figure 4A, myocilin was mainly detected extracellularly. As its expression was increased, however, the cellular distribution of myocilin shifted to intracellular accumulation and this gradually exceeded the extent of secretion from the cells. Therefore, at a higher expression level, myocilin was detected preferentially in the intracellular compartment (Figure 4B, upper panel). As a control, we monitored the movement of stromelysin, a well known secretory protein, which was secreted into the culture media regardless of its expression levels (Figure 4B, lower panel).

### Myocilin is retained in the lumen of the endoplasmic reticulum

**Figure 5:**
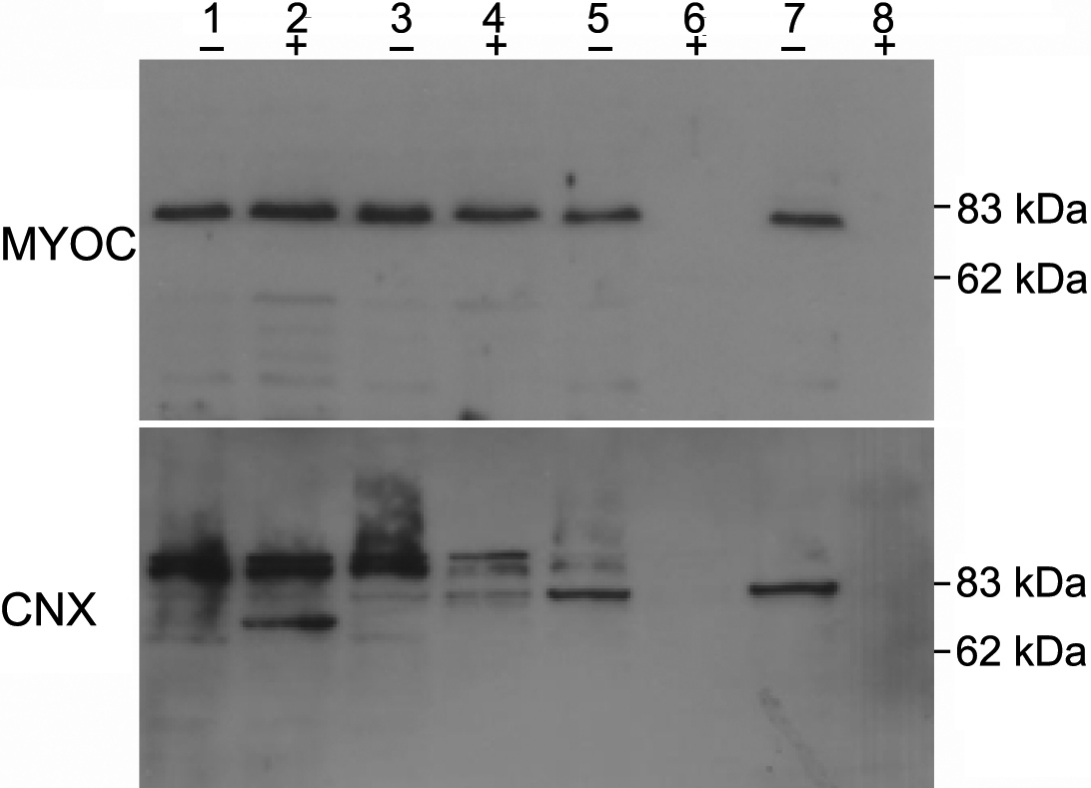
Determination of the topology of myocilin in the endoplasmic reticulum. The microsomal membrane was prepared from HTM cells transduced with Ad-myocilin-GFP. The membrane fractions were untreated (-) or treated with 1% Triton X-100 (+) followed by the addition of increasing concentrations of protease K (10 μg/ml in lane 1 and 2; 20 μg/ml in lane 3 and 4; 50 μg in lane 5 and 6; 100 μg lane 7 and 8). After digestion, the membrane was probed with anti-myocilin antibody (upper panel), stripped, and re-probed with anti-calnexin antibody (lower panel).

Because knowledge of the topology of a protein is necessary for elucidating its identity, we studied the topology of myocilin in the ER by the protease protection assay. We prepared microsomal vesicles from the HTM cells expressing myocilin-GFP and treated the membrane vesicles with increasing concentrations of proteinase K in the presence or absence of Triton X-100. Western blot analysis showed that myocilin was completely resistant to the protease in the absence of a detergent, but it was readily digested when the membrane was disrupted with Triton X-100 (Figure 5, upper panel), which is in accordance with a previous report [34]. We also probed the same blot with a monoclonal antibody against calnexin, a 90 kDa type I transmembrane protein primarily located in the ER. In the absence of a detergent, the full-length calnexin was all converted to a truncated form with its NH_2_-terminal fragment exposed to the cytoplasmic face of the membrane deleted while in the presence of a detergent, it was completely destroyed (Figure 5, lower panel). These data warrant membrane intactness and proteinase K activity, thereby demonstrating that myocilin is a luminal ER protein.

### Endoplasmic reticulum retention of myocilin occurs in a subset of cells

**Figure 6:**
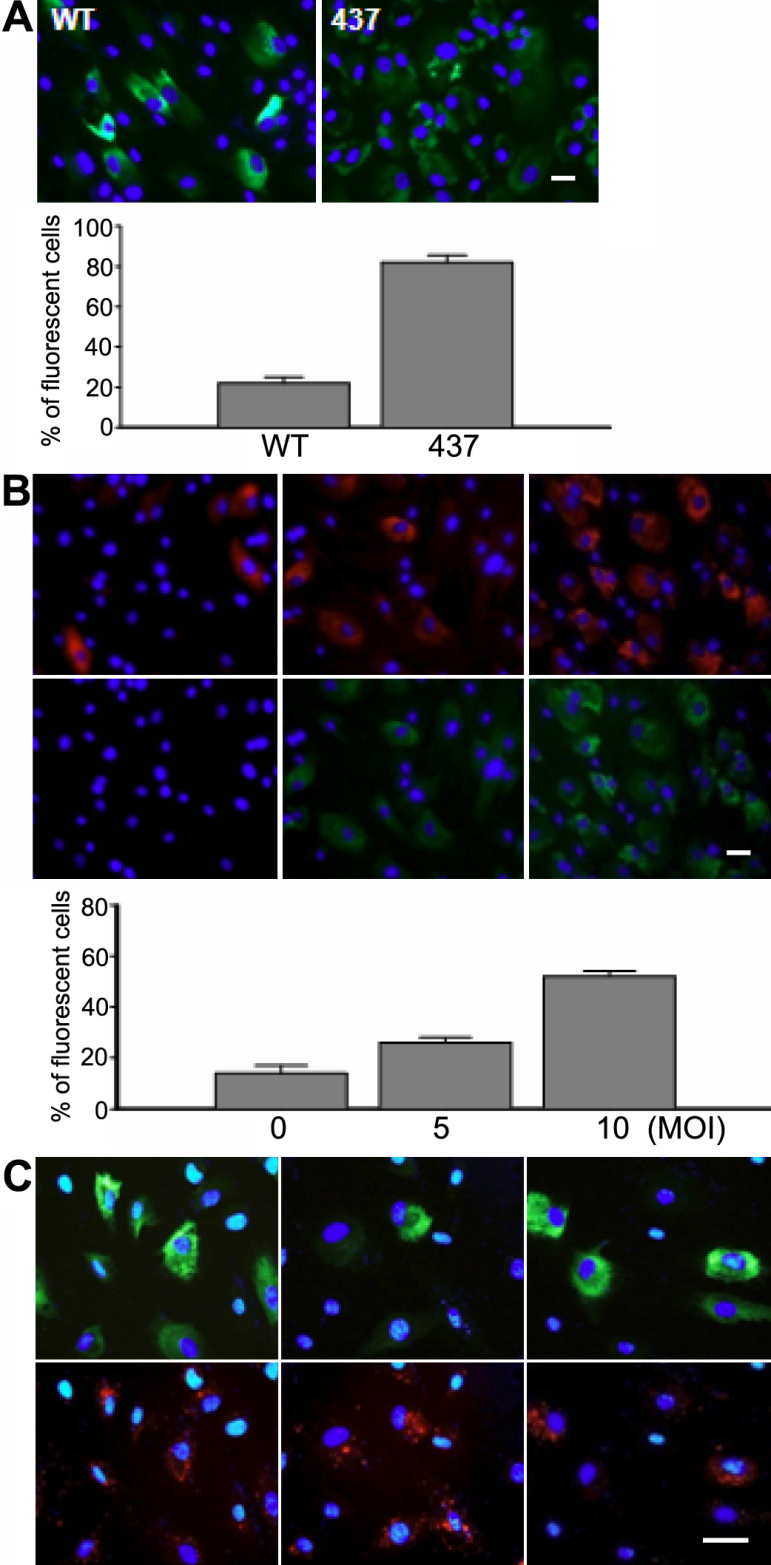
Fluorescence microscopic analysis of myocilin expression. **A**: HTM cells were transduced with Ad-myocilin-GFP (WT) or Ad-Y437H myocilin-GFP (437) at an MOI of 10 pfu and expressed for 48 h. After fixing and staining with DAPI, myocilin expression (green) and DAPI staining (blue) were merged. Representative images are shown of three independent experiments. The graph is plotted values showing the ratio of GFP-expressing cells to nontransduced cells. Data represents means±standard deviation from 200 cells. **B**: HTM cells were transduced with Ad-myocilin-FLAG at an MOI of 10 pfu followed by transduction of Ad-Y437H myocilin-GFP at an MOI of 0 pfu (left column), 5 pfu (middle column), and 10 pfu (right column). Myocilin-FLAG was detected with anti-FLAG antibody. Images of red fluorescence from myocilin-FLAG (upper row) or green fluorescence from Y437H myocilin-GFP (lower row) were merged with DAPI stains. Each column represents the same cells. Representative images are shown of three independent experiments. The graph is plotted values showing the ratio of red fluorescence-positive cells to nontransduced cells. Data represents means±standard deviation from 200 cells. **C**: HTM cells were transduced with red fluorescence (CM-Dil)-labeled Ad-myocilin-GFP. Green (upper panel) and red fluorescence (lower panel) overlapped with nuclear stains correspond to myocilin expression and cellular uptake of viral particles, respectively. Each column represents the same cells. Representative images are shown of three independent experiments. Bar: 50 μm.

Previously, no myocilin staining was reported even with DEX exposure in many TM cells and most Schlemm’s canal cells [35], ocular cells that along with TM cells constitute the outflow pathway. Our results consistently showed that the cultures transduced with Ad-myocilin-GFP demonstrated green fluorescence corresponding to myocilin expression in a restricted number of cells, with bright fluorescence noted occasionally (Figure 6A). This expression pattern of myocilin is easily distinguished from that of myocilin mutants such as myocilin with the Y437H mutation where the expression is discernable in nearly all cells, although the signal is less weak. To understand the distinct features of myocilin expression, we co-expressed wild-type myocilin with myocilin mutant with the Y437H mutation and thereby selectively inhibited the secretion of the former protein. As expected, inhibition of wild-type myocilin secretion resulted in a dramatic increase in the number of cells showing wild-type myocilin expression. Furthermore, the increase was proportional to the myocilin mutant expression (Figure 6B). These results suggest that wild-type myocilin is secreted immediately after its synthesis from most cells, making it hard to detect its expression. This explains why myocilin expression is not detected in many HTM cells. To better understand myocilin expression, HTM cells were transduced with Ad-myocilin-GFP that was covalently labeled with CM-Dil. In general, the level of a transgene expression in a cell is largely correlated with the degree with which the viral particles carrying the gene enter the cell. The correlation was observed only in about half of the transduced cells (Figure 6C, right row). Approximately 40% of the cells examined did not exhibit any myocilin expression as judged by the brightness of the green fluorescence of the GFP when compared to the extent of the viral uptake and as judged by the brightness of the red fluorescence of the CM-Dil (Figure 6C, middle row). The remaining 10% of cells showed myocilin expression without any prominent signs of viral infection (Figure 6C, left row). These results illustrate that the degree of intracellular myocilin accumulation is not solely determined by the extent of viral transduction. Perhaps it might also be influenced to a certain extent by individual cell-specific characteristics.

### Intracellular accumulation of myocilin leads to endoplasmic reticulum stress and reduction in cell proliferation

One explanation for the localization of myocilin to the ER is that myocilin is retained inside cells because it is not correctly folded or assembled in the ER. If this is true, myocilin may lead to, as misfolded or unfolded proteins usually do, an ER stress response such as unfolded protein response (UPR), which may be followed by apoptotic cell death [36,37]. In previous studies, we showed by western blot analysis and WST-1 (4-[3-(4-Iodophenyl)-. 2-(4-nitrophenyl)-2H-5-tetrazolio]-1,3-benzene disulfonate) cell proliferation assay that wild-type myocilin did not trigger ER stress and was not toxic to cells [16,17]. However, the biochemical assessments may have been inadequate in assaying such a protein as myocilin since they demonstrate only average effects of all the cells. To precisely evaluate the biological outcomes of myocilin expression, cellular responsiveness to myocilin accumulation must be examined in individual cells.

**Figure 7:**
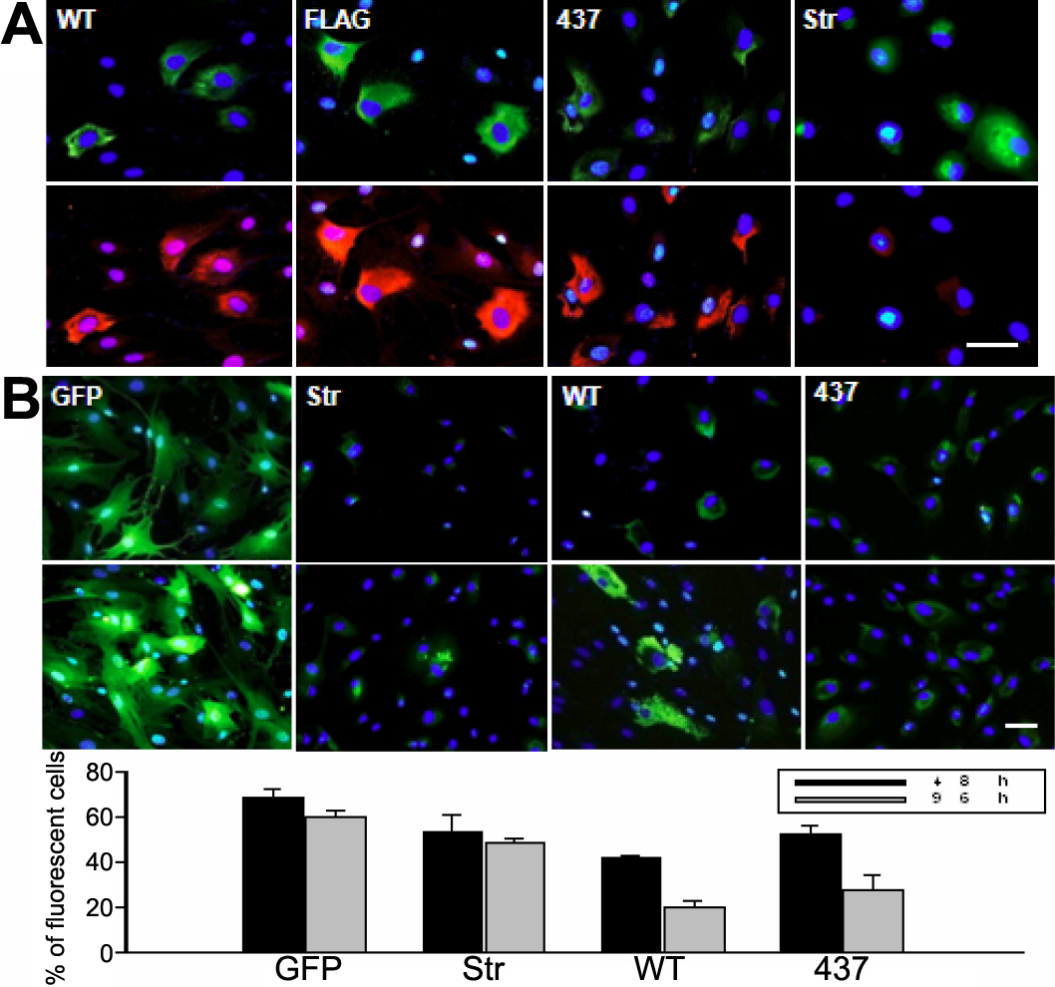
Evaluation of the potential cytotoxicity of myocilin expression. **A**: HTM cells were transduced with Ad-myocilin-GFP (WT), Ad-myocilin-FLAG (FLAG), Ad-Y437H myocilin-GFP (437), or Ad-stromelysin-GFP (Str) and stained for GRP78. FITC-conjugated anti-FLAG antibody and DAPI was used to stain myocilin-FLAG and nuclei, respectively. Each column represents the same cells. The staining patterns of each myocilin (green) and GRP78 (red) merged with DAPI stains are shown in the upper and lower rows, respectively. Representative images are shown of three independent experiments. **B**: HTM cells were transduced with Ad-GFP (GFP), Ad-stromelysin-GFP (Str), Ad-myocilin-GFP (WT), or Ad-Y437H myocilin-GFP (437) and cultured for 48 h (upper row) or 96 h (lower row). After fixing and staining with DAPI, the green fluorescence of GFP or GFP fusion proteins were merged with DAPI stains. Representative images are shown of three independent experiments. Bar: 50 μm. The graph is plotted values showing the ratio of GFP-expressing cells to nontransduced cells. Data represents means±standard deviation from 200 cells.

In the present study, therefore, we reexamined using the immunocytochemistry myocilin-induced ER stress response. The results showed that the UPR could be activated with wild-type myocilin expression as demonstrated by the upregulation of *GRP78* in the cells expressing myocilin-GFP but not in cells without myocilin-GFP expression. The same pattern of *GRP78* induction was observed in the cultures expressing myocilin-FLAG as well as the myocilin mutant such as myocilin with the Y437H mutation (Figure 7A). By contrast, the induction was not observed in cells expressing GFP-tagged stromelysin, demonstrating that the observations were not an artifact of the GFP tagging, protein overexpression, or the viral infection.

We then assessed by fluorescence microscopy the effect of myocilin expression on the proliferation of HTM cells. To this end, we transduced HTM cells with adenoviruses expressing GFP, stromelysin-GFP, myocilin-GFP, or Y437H myocilin-GFP and then calculated the ratio of cells expressing each transgene versus the cells that did not express it 48 h or 96 h later. In the cells transduced with Ad-GFP, the ratio was relatively constant. In the cells transduced with Ad-myocilin-GFP, however, the ratio at the 96 h post-transduction was approximately half of that at 48 h post-transduction. A similar reduction in the ratio was observed with cells transduced with Ad-Y437H myocilin-GFP (Figure 7B). These results suggest that the replication of the cells expressing wild-type or mutant myocilins was significantly inhibited compared to the cells that were not transduced. These findings indicate that the expression of not only mutant myocilin but also wild-type myocilin appears to be toxic to cells.

## Discussion

In this study, we were interested in understanding the mechanism by which myocilin is localized to both the ER and extracellular compartments. The most common mechanism by which products of a single gene are localized to more than one cellular compartment is for each protein to be generated with different topogenic information [30]. Hence, we first tried to search for the presence of myocilin isoforms that are topogenically different but failed to find such differences. Essentially, intracellular myocilin does not differ from its extracellular counterpart.

The present study confirmed the previous observations that both intracellular and extracellular myocilins are resolved on SDS–PAGE into glycosylated or non-glycosylated forms and that these myocilins can be further separated on 2D-PAGE into multiple spots with different pIs [22,23,33]. Strictly speaking, however, the glycosylated and non-glycosylated myocilins do not fulfill our criteria for myocilin isoforms with respect to cellular trafficking because both are equally secreted. Similarly, the myocilin isoforms identified by 2D-PAGE analysis also do not meet our criteria because most of them are present intracellularly and extracellularly. Although it was reported that myocilin undergoes post-transcriptional modification giving rise to deleted forms [38], this finding was not informative in that the event was examined only with intracellular myocilin. Furthermore, this finding has not been confirmed as yet by others. It was also reported that the Met-Arg-Phe-Phe-Cys-Ala sequence (amino acids 1–6) was obtained for the higher molecular mass of myocilin doublet and the Arg-Thr-Ala-Gln-Leu sequence (amino acids 33–37) was for the lower molecular mass of myocilin doublet [32]. However, these results are also not informative for the same reasons. Recently, Escribano et al. [26,34] have shown that myocilin undergoes an intracellular endoproteolytic processing, resulting in a 35 kDa fragment. In our experience, however, the 35 kDa protein was occasionally observed even when myocilin was overexpressed (data not shown). Moreover, the fragment was detected in both the cells and culture media, suggesting that it is not the ER specific or extracellular compartment specific protein. Except for these few reports, there is no published data regarding alternative RNA splicing or different translational initiation, the most common mechanisms that enable a single gene to differentially express multiple types of topogenic information [30]. The early description that *MYOC* is composed of only three different sized exons further suggests that alternative RNA splicing seldom, if ever, occurs in this gene [32,39]. Taken together, our data and those from other investigators strongly suggest that myocilin isoforms that are different in both their primary structure and topogenic information do not exist.

In this study, we demonstrated that myocilin was not completely secreted but accumulated in the lumen of the ER over time. Many luminal ER proteins have at their COOH-terminus a tetrapeptide Lys-Asp-Glu-Leu (KDEL) sequence and display posttranslational modifications that are performed by enzymes in the Golgi apparatus, demonstrating that they escape the ER at least once during their lifetime [40,41]. However, myocilin does not have the KDEL sequence. In addition, myocilin did not respond to Endo D digestion, suggesting that it never leaves the ER. Furthermore, myocilin was not evenly expressed in cells transduced with the adenovirus expressing myocilin. Therefore, these results do not indicate that myocilin is a cytosolic protein destined for localization in the ER.

Although most *MYOC* mutations that are associated with glaucoma are missense substitutions [7-9], myocilin with these mutations usually manifest phenotypes such as Triton insolubility [42], formation of intracellular aggregates [14,16-18], and blockage of secretion [14,15,17]. This suggests that myocilin is prone to misfolding. It has been reported that a very high proportion (30%) of all newly synthesized proteins are defective, but they are apparently degraded by proteasomes shortly after their synthesis [43]. The present study showed that such was not the case with myocilin in that it was relatively resistant to proteasomal degradation. The accumulation of such proteins as myocilin that are misfolded and not degraded may be potentially detrimental to cells. We observed that the cells expressing myocilin always expressed GRP78, the master regulator of UPR as well as ER stress [36]. This observation suggests that intracellular myocilin is a protein similar to its mutant forms where it is localized to the ER by default and at this location is potentially cytotoxic.

Recent studies have investigated transgenic mice homozygous for *MYOC* and estimated the steady-state level of myocilin in the AH of the mice, demonstrating that mice overexpressing the wild-type myocilin did not develop elevated IOP or glaucoma [44,45]. These results suggest that increasing the amounts of myocilin alone is not sufficient to cause glaucoma. However, these findings do not mean that intracellular myocilin itself is not harmful to cells. Therefore, one might speculate that various published biological outcomes associated with myocilin [46,47] may be attributed to the potential cytotoxicity of myocilin expression.

In summary, the present study investigated whether intracellular wild-type myocilin is a true ER resident protein and showed that myocilin is likely a mistargeted protein rather an ER protein. Therefore, the role myocilin plays in the development of POAG may be greater than initially predicted.
